# Daily torpor reduces the energetic consequences of microhabitat selection for a widespread bat

**DOI:** 10.1002/ecy.3677

**Published:** 2022-04-11

**Authors:** Jesse M. Alston, Michael E. Dillon, Douglas A. Keinath, Ian M. Abernethy, Jacob R. Goheen

**Affiliations:** ^1^ Program in Ecology, Department of Zoology and Physiology University of Wyoming Laramie Wyoming USA; ^2^ Wyoming Natural Diversity Database University of Wyoming Laramie Wyoming USA; ^3^ Center for Advanced Systems Understanding (CASUS) Görlitz Germany; ^4^ Wyoming Ecological Services Field Office United States Fish and Wildlife Service Cheyenne Wyoming USA

**Keywords:** Bayesian hierarchical models, climate change, daily torpor, fringed myotis (*Myotis thysanodes*), temporal heterothermy, thermal ecology, VHF telemetry

## Abstract

Homeothermy requires increased metabolic rates as temperatures decline below the thermoneutral zone, so homeotherms typically select microhabitats within or near their thermoneutral zones during periods of inactivity. However, many mammals and birds are heterotherms that relax internal controls on body temperature and go into torpor when maintaining a high, stable body temperature, which is energetically costly. Such heterotherms should be less tied to microhabitats near their thermoneutral zones and, because heterotherms spend more time in torpor and expend less energy at colder temperatures, heterotherms may even select microhabitats in which temperatures are well below their thermoneutral zones. We studied how temperature and daily torpor influence the selection of microhabitats (i.e., diurnal roosts) by a heterothermic bat (*Myotis thysanodes*). We (1) quantified the relationship between ambient temperature and daily duration of torpor, (2) simulated daily energy expenditure over a range of microhabitat temperatures, and (3) quantified the influence of microhabitat temperature on microhabitat selection. In addition, warm microhabitats substantially reduced the energy expenditure of simulated homeothermic bats, and heterothermic bats modulated their use of daily torpor to maintain a constant level of energy expenditure across microhabitats of different temperatures. Daily torpor expanded the range of energetically economical microhabitats, such that microhabitat selection was independent of microhabitat temperature. Our work adds to a growing literature documenting the functions of torpor beyond its historical conceptualization as a last‐resort measure to save energy during periods of extended or acute energetic stress.

## INTRODUCTION

The thermal environments in which organisms live strongly influence metabolic rates (Brown et al., 2004; Huey & Stevenson, 1979; Pörtner & Knust, 2007). Among homeotherms—which regulate body temperature internally within a narrow range to optimize physiological processes—metabolic heat production is tightly regulated in response to variation in temperature in the surrounding environment (i.e., ambient temperature; Lowell & Spiegelman, 2000). Controlling body temperature therefore requires increased energy expenditure by homeotherms when ambient temperatures depart from the thermoneutral zone (i.e., the range of ambient temperatures in which homeotherms can maintain body temperature with minimal metabolic effort; McNab, 2002).

Although the influence of ambient temperature on metabolism in homeotherms is understood relatively well, many animals are heterotherms that can temporarily or partially allow body temperature to track ambient temperature (Withers et al., 2016). Heterothermy is common among mammals and birds (Geiser, 2004; Geiser & Ruf, 1995; McKechnie & Mzilikazi, 2011; Ruf & Geiser, 2015) and can reduce energy expenditure during both hot and cold periods (Boyles et al., 2016; Nowack et al., 2017; Reher & Dausmann, 2021; Stawski & Geiser, 2012). As ambient temperatures depart from the thermoneutral zone, heterotherms can relax internal controls on metabolism; this physiological response allows body temperature to track ambient temperature and reduce the energetic costs of maintaining stable body temperatures outside the thermoneutral zone (Levesque et al., 2016). Heterotherms often achieve this by entering torpor, a hypometabolic state of inactivity that ranges from daily torpor (periods of brief torpor that may last less than an hour) to hibernation (sustained periods of torpor that can last for most of a year; Hoelzl et al., 2015; Ruf & Geiser, 2015).

**FIGURE 1:**
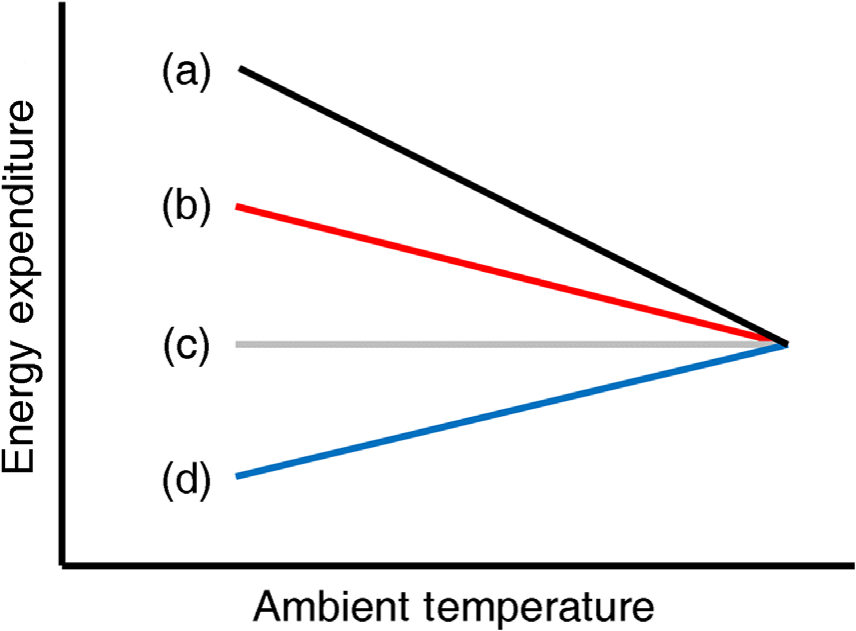
Heuristic diagram outlining the potential energetic benefit to an individual bat of using periodic bouts of daily torpor rather than remaining in homeothermy at ambient temperatures below the thermoneutral zone. This diagram is similar to a classic Scholander curve except for one detail: while a Scholander curve illustrates metabolic rate or energy expenditure at a constant ambient temperature and physiological state (i.e., either homeothermy or torpor) in laboratory conditions, this diagram illustrates energy expenditure when ambient temperature and physiological state vary through time, as they do in field conditions. Specifically, this diagram assumes that (1) bats use more daily torpor when it is cold than when it is warm, (2) ambient temperatures vary over the course of the day, and (3) ambient temperatures below the thermoneutral zone are more prevalent than ambient temperatures above the thermoneutral zone (please refer to Cunningham et al., 2021). Each hypothetical relationship would result in a different pattern of microhabitat selection by animals seeking to minimize energy expenditure during periods of inactivity. The black (a) line represents energy expenditure over a day while maintaining homeothermy 100% of the time (i.e., never using daily torpor). The red (b), gray (c), and blue (d) lines indicate energy expenditure over a day while using some amount of daily torpor. For all three relationships, daily torpor provides energy savings (i.e., the difference between the black and other lines), and these savings are most pronounced at colder ambient temperatures. Line (b), for bats that use daily torpor, energy expenditure increases at colder ambient temperatures because, while some energy is saved from employing daily torpor, maintaining homeothermy is more costly at colder than at warmer ambient temperatures. A bat exhibiting this relationship should select warm microhabitats to reduce energy use. Line (c), for bats that use daily torpor, energy expenditure is stable across a wide range of ambient temperatures because the energy saved from employing daily torpor matches (and thus offsets) the increase in energy expended to maintain homeothermy at colder temperatures. A bat exhibiting this relationship should not benefit from selecting either warm or cool microhabitats, and should thus select neither warm nor cool microhabitats. Line (d), for bats that use daily torpor, energy expenditure decreases at colder ambient temperatures because relatively more energy is saved from using daily torpor even as maintaining homeothermy is more costly at colder than at warmer ambient temperatures. A bat exhibiting this relationship should select cool microhabitats to reduce energy use

Reducing energy expenditure during extended periods of resource scarcity was historically believed to be the primary function of torpor, but biologists are increasingly interested in alternative functions of torpor, particularly over shorter timescales (Nowack et al., 2017). For example, how do animals use daily torpor to maintain energy balances over short timescales, and how does temperature influence that process? Heterotherms use daily torpor more as ambient temperatures decline below the thermoneutral zone (Chruszcz & Barclay, 2002; Rambaldini & Brigham, 2008; Solick & Barclay, 2006; Song & Geiser, 1997), but it is unclear how this tendency translates to differences in energy expenditure across differences in temperature. For a given period of time, total energy expenditure for heterotherms depends on (1) the duration and frequency of bouts of daily torpor, (2) their thermal environments (i.e., ambient temperatures and factors such as wind, humidity, solar radiation, and contact with substrates that affect heat transfer), and (3) the difference in metabolic rates between daily torpor and homeothermy in a given thermal environment. If a heterotherm uses no daily torpor, energy expenditure will be substantially higher at colder temperatures than at warmer temperatures (Figure 1, line a). If a heterotherm uses some daily torpor, energy expenditure might be lower than in Figure 1, line a, but still increase as ambient temperatures fall below the thermoneutral zone. Even though heterotherms save energy by using daily torpor some of the time, declines in energy expenditure from using daily torpor more when it is cold do not fully compensate for the increased energetic costs of maintaining homeothermy in colder ambient temperatures during periods when animals are not in torpor (Figure 1, line b). In this scenario, periodic bouts of daily torpor dampen, but do not completely offset, increases in energy expenditure during periods of homeothermy at cold ambient temperatures. Alternatively, it is possible that energy expenditure by heterotherms is stable through a wide range of ambient temperatures, because energy savings from spending progressively more time in daily torpor at progressively colder ambient temperatures closely match the increases in energy expenditure from maintaining homeothermy at colder ambient temperatures (Figure 1, line c). Finally, as ambient temperatures decline, the energetic savings from daily torpor could more than offset the increased energy expenditure necessary to maintain homeothermy (Figure 1, line d).

Such relationships between ambient temperature and energy expenditure have cascading repercussions for other aspects of an animal's life. For example, because survival and reproduction require that energy intake equals or exceeds energy expenditure, operating in ambient temperatures outside the thermoneutral zone can reduce fitness over time (Angilletta et al., 2010; Boyles et al., 2011). Animals seeking to maximize fitness therefore often select microhabitats with temperatures that minimize energy expenditure (e.g., Alston et al., 2020; Freitas et al., 2016; Huey, 1991; Sarmento et al., 2019). Homeotherms have relatively fixed relationships between ambient temperature and metabolic rate, and therefore often consistently select microhabitats to maintain optimal body temperatures with little metabolic effort (e.g., Courbin et al., 2017; Poole et al., 2016; Sarmento et al., 2019). In contrast, looser relationships between ambient temperature and metabolic rate for heterotherms may allow heterotherms to select microhabitats with less regard to ambient temperature, or even to prefer microhabitats that might be colder than optimal for homeotherms. For example, heterothermic Australian owlet‐nightjars (*Aegotheles cristatus*) preferentially roost in colder, less thermally stable tree cavities, whereas homeothermic cavity‐nesting birds typically select warmer, more thermally stable tree cavities (Doucette et al., 2011). Empirical data on the thermal characteristics of microhabitat selection by heterotherms are rare, however, particularly for free‐ranging animals.

Uncertainty surrounding the form and strength of relationships between ambient temperature and energy expenditure limits our understanding of temperature‐driven microhabitat selection by heterotherms. For an animal attempting to minimize energy expenditure during periods of inactivity, each of the hypothetical relationships between energy expenditure and ambient temperature in Figure 1 would result in a different pattern of microhabitat selection. A heterotherm exhibiting the relationship shown by the red (b) line in Figure 1 should select warm microhabitats to save energy, similar to homeotherms (a). A heterotherm exhibiting the relationship shown by the gray (c) line in Figure 1 should not select microhabitats based on their thermal characteristics. This pattern of microhabitat selection would diverge from the pattern followed by homeotherms. A heterotherm exhibiting the relationship shown by the blue (d) line in Figure 1 should select cool microhabitats to save energy, the opposite of the pattern followed by homeotherms. Empirical tests of the influence of ambient temperature on energy expenditure are therefore needed to understand how ambient temperature drives microhabitat selection for heterotherms.

**FIGURE 2:**
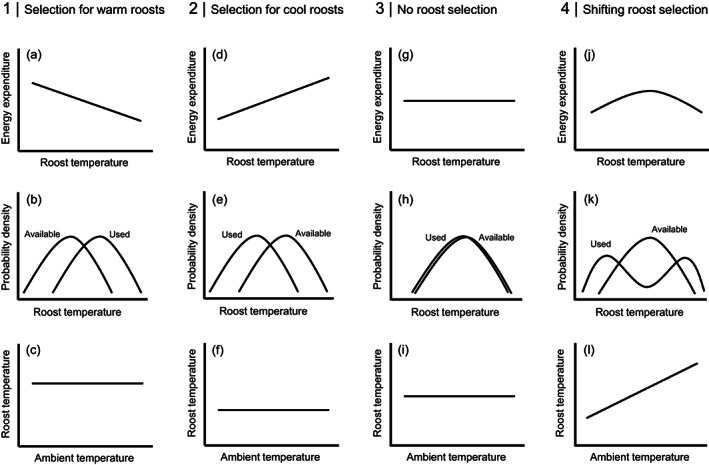
Four competing sets of predictions of microhabitat selection by a heterothermic bat. Each column represents one of four sets of predictions, and each row represents a statistical relationship consistent with the predictions. In column 1, energy expenditure over the course of a day is higher in cool microhabitats than in warm microhabitats (a). In response, bats select warm microhabitats to minimize energy expenditure during the day (b). In this scenario, there should be no directional relationship between ambient temperature and microhabitat temperature (i.e., bats always select warm microhabitats regardless of ambient temperature; (c)). In column 2, energy expenditure over the course of a day is higher in warm microhabitats than in cool microhabitats (d). In response, bats select cool microhabitats to minimize energy expenditure during the day (e). In this scenario, there should be no directional relationship between ambient temperature and microhabitat temperature (i.e., bats always select cool microhabitats regardless of ambient temperature; (f)). In column 3, energy expenditure over the course of a day is constant across microhabitats of all temperatures (because bats can adaptively use daily torpor so that temperatures within microhabitats have little influence on overall energy expenditure; (g)). Because energy expenditure is consistent across microhabitats of all temperatures, bats do not select microhabitats due to temperatures within microhabitats (h). In this scenario, there is no relationship between ambient temperature and temperature of used microhabitats (i.e., bats never select microhabitats due to temperatures within microhabitats, regardless of ambient temperature; (i)). In column 4, energy expenditure peaks at intermediate microhabitat temperatures at which bats use relatively little daily torpor, but the costs of maintaining homeothermy are relatively high (j). In response, bats select cool microhabitats on cool days and warm microhabitats on warm days (k) because daily torpor saves more energy in cool microhabitats than in warm microhabitats. In this scenario, the relationship between ambient temperature and microhabitat temperature should be positive (i.e., bats select warmer microhabitats on warmer days; (l))

We sought to understand how ambient temperature influences energy expenditure, and how energy expenditure in turn influences microhabitat selection (i.e., selection of diurnal roosts) in a bat that is widely distributed throughout western North America (fringed myotis, *Myotis thysanodes*). Like other bats that inhabit temperate latitudes, fringed myotis are heterotherms that are believed to select microhabitats to minimize energy expenditure during diurnal periods of inactivity (Ruczyński, 2006; Sedgeley, 2001; Willis & Brigham, 2005). At temperate latitudes, temperatures within structures commonly used as roosts by bats (e.g., rock crevices, tree cavities, and abandoned buildings) can vary substantially throughout the day and year, and ambient temperature influences the amount of time that bats spend in daily torpor. Like other heterotherms, bats spend more time in daily torpor when it is cold than when it is hot (Chruszcz & Barclay, 2002; Rambaldini & Brigham, 2008; Solick & Barclay, 2006). We hypothesized that the differences in energy expenditure arising from variation in temperature between microhabitats drive patterns of microhabitat selection (i.e., bats select microhabitats that minimize energy expenditure). Specifically, we weighed evidence for four competing sets of predictions (Figure 2):

Prediction Set 1: Bats select warm microhabitats regardless of ambient temperature. In this scenario, energy expenditure during the day should be higher in cool microhabitats than in warm microhabitats (Figure 2a), because the energetic benefits from being warmer when bats are maintaining homeothermy outweigh the energetic costs of spending less time in daily torpor. If this is the case, bats should select microhabitats that are warmer compared with available microhabitats on the landscape (Figure 2b); this pattern of selection should be consistent, regardless of ambient temperature during the day (Figure 2c).

Prediction Set 2: Bats select cool microhabitats regardless of ambient temperature. In this scenario, energy expenditure during the day should be higher in warm microhabitats than in cool microhabitats (Figure 2d), because the energetic benefits from spending more time in daily torpor outweigh the energetic costs of being colder when bats are maintaining homeothermy. If this is the case, bats should select microhabitats that are cooler compared with available microhabitats on the landscape (Figure 2e); this pattern of selection should be consistent regardless of ambient temperature during the day (Figure 2f).

Prediction Set 3: Bats do not alter microhabitat selection as ambient temperatures change. In this scenario, energy expenditure during the day is roughly equal across microhabitats of all temperatures (Figure 2g). This could occur if bats modulate use of daily torpor, such that temperatures within microhabitats have little influence on overall energy expenditure. In this case, bats should select microhabitats that are similar in temperature to available microhabitats on the landscape (Figure 2h), and this pattern of selection should be consistent regardless of ambient temperature during the day (Figure 2i).

Prediction Set 4: Bats select cool microhabitats on cool days and warm microhabitats on warm days (shifting microhabitat selection). In this scenario, energy expenditure is lower in cool microhabitats than in warm microhabitats on cool days, lower in warm microhabitats than in cool microhabitats on warm days, and consistently higher in microhabitats at intermediate ambient temperatures (Figure 2j). This may arise because of threshold effects from a non‐linear relationship between ambient temperature and use of daily torpor. Namely, a threshold may exist above which homeothermy requires relatively little energy, even as bats spend little time in daily torpor, but below which bats save a substantial amount of energy by using daily torpor. Near the threshold, however, bats may use relatively little daily torpor even as maintaining homeothermy is relatively energetically costly. In this case, bats should select microhabitats that are roughly the same temperature on average as available microhabitats on the landscape (although the distribution may be bimodal; Figure 2k), and temperatures in microhabitats should be positively correlated with ambient temperature (Figure 2l).

## METHODS

### Study area and species

We conducted our study during the summers of 2017 and 2018 on Jewel Cave National Monument (43°45′ N, 103°45′ W) and surrounding areas of the Black Hills National Forest in South Dakota, USA. Our study area is described in Alston et al. (2019). Mean monthly summer high temperatures range between 22–27°C and mean monthly summer precipitation ranges between 60–80 mm (Western Regional Climate Center, 2018). Open ponderosa pine (*Pinus ponderosa*) forests dominate, with Rocky Mountain juniper (*Juniperus scopulorum*) and quaking aspen (*Populus tremuloides*) occurring locally. Forests are actively managed to prevent wildfire, and those managed by the US Forest Service and private landowners also undergo intensive logging. Forests form a mosaic with northern mixed‐grass prairie where a large stand‐replacing fire occurred in 2000. A large network of caves lies underground and the landscape exhibits substantial topographic relief in the form of intersecting canyon systems and rock outcrops.

Fringed myotis primarily roost in rock crevices, tree cavities, and under the sloughing bark of dead trees, although they have occasionally been documented roosting in caves, mines, and buildings (Hayes & Adams, 2015; Lacki & Baker, 2007). They forage in the forest canopy and riparian areas (O'Farrell & Studier, 1980). We chose to focus on males because sex ratios of bats in the Black Hills are heavily (>90%) male‐biased (a common pattern in high‐elevation areas; Barclay, 1991; Cryan et al., 2000; Senior et al., 2005), because male *M. thysanodes* usually roost solitarily (O'Farrell & Studier, 1980), and because male bats maintain consistent patterns of daily torpor use throughout the reproductive season (unlike females, which alter patterns of daily torpor use at different stages of reproduction; Chruszcz & Barclay, 2002; Dzal & Brigham, 2013; Johnson & Lacki, 2014).

### Capture and VHF telemetry

We used mist nets to capture bats over permanent and semipermanent water sources (e.g., springs, stock tanks, and stock ponds). From June through August of 2017 and 2018, we netted on 20 and 49 nights, respectively, at 15 water sources. We opened mist nets at civil sunset and closed them after 5 h or during inclement weather.

We fixed temperature‐sensitive very high frequency (VHF) transmitters (LB‐2XT model 0.28/0.33 g; Holohil Systems Ltd., Carp, ON, Canada) between the scapulae of adult male fringed myotis with latex surgical adhesive (Osto‐Bond, Montreal Ostomy, Montreal, QC, Canada). The transmitters measure and transmit data on skin temperature—an accurate proxy for core body temperature—of individual bats, enabling researchers to delineate bouts of daily torpor (Barclay et al., 1996; Chruszcz & Barclay, 2002; Stawski & Geiser, 2010). All transmitters weighed <5% of the mass of the bat (Aldridge & Brigham, 1988). We tracked bats to diurnal roosts each day that the transmitters were active and installed VHF data loggers (SRX800‐D1; Lotek Wireless Inc., Newmarket, ON, Canada) that collected and recorded data transmitted by the VHF transmitters. All protocols were approved by the University of Wyoming and National Park Service Animal Care and Use Committees and met guidelines approved by the American Society of Mammalogists for research on wild mammals (Sikes and the Animal Care and Use Committee of the American Society of Mammalogists, 2016).

### Energetic modeling

To quantify use of daily torpor, we used skin temperature data from individual bats to delineate bouts of daily torpor from data logger readings that captured full days (i.e., from entry in the morning to exit in the evening). This was a fraction of total days in which we located bats in microhabitats, because bats typically were not located until after they entered microhabitats for the day. We defined torpor as beginning when skin temperature dropped below the lowest skin temperature of bats maintaining homeothermy during a day and as ending when skin temperature began a steep rise that led to bats re‐entering homeothermy or leaving a microhabitat (as recommended by Barclay et al., 2001; Appendix S1: Figure S1). Because fat reserves and body mass can substantially alter the amount of time spent in daily torpor (Stawski & Geiser, 2010; Vuarin et al., 2013; Wojciechowski et al., 2007), we also controlled for the effect of body mass of each individual at time of capture on the duration of daily torpor. We then used the modeling software “Stan” (Carpenter et al., 2017) via the R package “brms” (v2.13.0; Bürkner, 2017) to build a linear Bayesian hierarchical model to quantify the influence of ambient temperature and body mass on the duration of daily torpor, while accounting for non‐independence among data points collected from the same individual. The model included three chains run for 12,000 iterations (2000 iterations of warm‐up and 10,000 iterations of sampling). We assessed chain convergence using the Gelman–Rubin diagnostic (*Ȓ*) and precision of parameter estimation using effective sample size. *Ȓ* <1.01 and effective sample sizes >10,000 represent acceptable convergence and parameter precision (Gelman et al., 2013; Kruschke, 2015). We used the R packages “loo” (v2.2.0; Vehtari et al., 2017) and “bayesplot” (v1.7.2; Gabry et al., 2019) to visually assess model diagnostics.

To quantify energy expenditure in bats, we combined published estimates of metabolic rates of fringed myotis as a function of temperature (Studier & O'Farrell, 1976) and the linear model of the influence of ambient temperature on daily torpor use to simulate the influence of temperature within microhabitats on energy expenditure. Specifically, we simulated minute‐by‐minute energy expenditure by bats in each used microhabitat between 4:45 AM and 9:00 PM (typical entry and exit times for bats in our study) on each day over the duration of our study period. We modeled use of daily torpor as a function of decision rules that reflect daily torpor use observed over the course of our study (raw data presented in Appendix S1: Table S1). Specifically, we assumed that bats entered torpor immediately upon entering microhabitats, exited torpor after an interval determined by temperatures within microhabitats, and remained in homeothermy for the rest of the time spent in the microhabitat except for a shorter bout of torpor in the evening. We further assumed that bats would use 86.9% of the duration of torpor in the morning and 13.1% in the afternoon unless the afternoon bout of torpor would be less than 30 min in duration, in which case 100% of the day's torpor would occur in the morning period. We also assumed that the mean duration of torpor that we observed would be used in the baseline “average” microhabitat, with the duration of torpor in warmer and cooler microhabitats determined by the slope of the modeled relationship between ambient temperature and daily torpor use described in the above paragraph. To account for uncertainty in our estimate of the slope of the relationship between ambient temperature and daily torpor use, for each microhabitat on each day we randomly drew a different slope estimate for this relationship from the posterior distribution of slope estimates from the model described in the prior paragraph.

### Microhabitat characterization

To characterize rock microhabitats, we collected data for 31 microhabitats and 62 randomly sampled available (i.e., unused by bats in our study) microhabitats. From this point forward, we distinguish between “used microhabitats” and available but unused “available microhabitats.” We identified available rock microhabitats in two ways: at each used microhabitat, we (1) located the nearest rock crevice large enough to hold a bat, and (2) generated a paired point in a random cardinal direction a random distance between 100–300 m away, then located the nearest rock crevice large enough to hold a bat.

To characterize tree microhabitats, we collected data for nine used microhabitats and 36 randomly sampled available microhabitats. We identified available tree microhabitats in two ways: at each used microhabitat, we (1) located the nearest dead tree and selected the nearest cavity large enough to hold a bat, and (2) generated a paired point in a randomly determined distance between 100–300 m away, in a randomly determined (cardinal) direction, then located the nearest tree cavity large enough to hold a bat. For each available point, we placed data loggers in two locations: one in a cavity in the trunk and one underneath sloughing bark. We defined available trees as any dead tree with a visible defect (e.g., sloughing bark or cavities) sufficiently large to hold a bat. This description fit every tree in which we found a roosting bat.

In summer 2018, we monitored temperatures within both used and available microhabitats using data loggers (Model MX2201; Onset Computer Corporation, Bourne, MA, USA). The first data loggers were deployed on 17 July 2018, and the last data logger was removed on 8 October 2018. This period of time includes the full range of daily high temperatures occurring during the active season for bats at our study site. During data logger deployment and opportunistically thereafter, we checked microhabitats with data loggers inside them for the presence of bats. We sometimes found bats in used microhabitats, but we never found bats in available microhabitats. When we found bats in used microhabitats, we waited to deploy data loggers until there was no bat within the microhabitat.

To quantify the thermal characteristics of each microhabitat, we calculated the mean temperature within each microhabitat for periods between 4:45 AM and 9:00 PM, which corresponds to the period in which a bat is likely to be within a microhabitat (Appendix S1: Table S1). To control for potential confounding variables, we also calculated the timing of peak temperature in all microhabitats (because if two microhabitats have the same mean temperature but peak in temperature at different times, the microhabitat with the later peak will have cooler temperatures in the morning when bats use daily torpor most), and the standard deviation of temperature during the day (because stability in microhabitat temperature can be an important factor in microhabitat selection by bats; Sedgeley, 2001). To quantify the timing of the daily temperature peak, we located the peak temperature in each microhabitat for each day and calculated the mean time of day at which this occurred over our study period. To quantify thermal stability in microhabitats, we calculated the standard deviation of temperatures between 4:45 AM and 9:00 PM in each microhabitat for each day and calculated the mean daily standard deviation over our study period. To ensure consistency, we only calculated these values for the period between 28 July and 31 September (a period in which all data loggers were actively logging temperatures, and in which average daily high temperatures corresponded to the range a bat might be exposed to during the active season in our study area).

We used the R statistical software environment (R Core Team, 2020) to quantify differences between used and available microhabitats. To determine whether bats selected cooler microhabitats than those available, we used the modeling software “Stan” (Carpenter et al., 2017) via the R package “brms” (v2.13.0; Bürkner, 2017) to build a binomial‐family Bayesian model to quantify the influence of mean temperature within microhabitats, the timing of daily peaks in temperature within microhabitats, and the standard deviation of temperatures within microhabitats on microhabitat selection. The model included three chains run for 12,000 iterations (2000 iterations of warm‐up and 10,000 iterations of sampling). We assessed chain convergence using *Ȓ* and precision of parameter estimation using effective sample size. We checked predictive performance with receiver operating curve analysis using the R package “pROC” (v1.16.2; Robin et al., 2011) and used the R package “bayesplot” (v1.7.2; Gabry et al., 2019) to visually assess binned residual plots.

## RESULTS

We tracked 46 bats to 107 roosts (93 in rock crevices and 14 in trees). All roosts were located in shallow (<30 cm) crevices or cavities, and bats or VHF transmitters were usually visible from outside of the roost. We collected 27 full days of skin temperature data from seven bats. Data from 16 data loggers within microhabitats (3 used rock, 13 available rock, 1 available tree) could not be collected because they were not relocated or were dislodged from microhabitats. We therefore excluded these data from analyses, leaving a total of 121 (77 rock, 44 tree) data loggers that collected data on temperatures within microhabitats.

**FIGURE 3:**
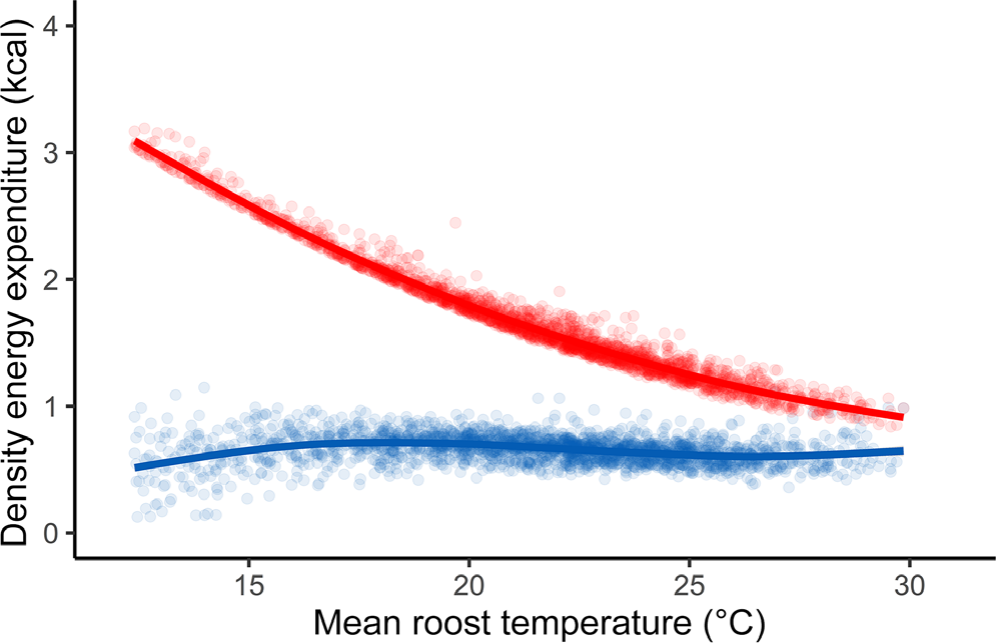
Results of our simulation of daily energy expenditure by fringed myotis over the range of temperatures observed in used microhabitats. Each point represents 1 simulated day. The red points represent estimated daily energy expenditure if bats never used daily torpor. The blue points represent our estimate of energy expenditure over the course of a day if part of the day is spent in daily torpor (with daily duration of torpor a function of daily ambient temperature as observed during our study). The lines represent locally estimated scatterplot smoothing (LOESS) regressions of the relationship between microhabitat temperature and daily energy expenditure. Estimates of daily energy expenditure incorporating observed bat behavior are steady across all temperatures observed within microhabitats during our study. The blue points in this figure correspond to the top row in Figure 2, and are most closely matched by Figure 2g

Use of daily torpor stabilized daily energy expenditure across the range of microhabitat temperatures observed during our telemetry study. In our model of the effect of ambient temperature on daily torpor duration, 95% credible intervals for the effect of mean ambient temperature over the course of the day on daily torpor duration did not cross zero (parameter estimate: −37.3 min; 95% credible intervals: −63.8 to −12.3 min), indicating that bats spent approximately 37 min less in torpor per day for each additional 1°C in daily mean ambient temperature between 4:45 AM and 9:00 PM (Appendix S1: Figure S2). When incorporated into our simulation of bat energy expenditure over the course of a typical day, this estimate of the relationship between ambient temperature and daily torpor use led to similar estimates of energy expenditure across temperatures within used microhabitats (Figure 3; blue points). Daily energy expenditure was comparable across the range of observed mean temperatures in all microhabitats (Figure 3; blue line). Our estimates for energy expenditure using observed bat behavior were always substantially lower and less variable than our estimates for energy expenditure if bats had remained in homeothermy all day (Figure 3; red points). Bats that remained in homeothermy would expend substantially more energy in cool microhabitats than in warm microhabitats.

Overall, temperatures in both rock and tree microhabitats were similar, although microhabitats in trees were slightly cooler and less stable than microhabitats in rocks. We therefore pooled rock and tree microhabitats in microhabitat selection analyses, but we reported descriptive statistics for each type of microhabitat in Appendix S1.

**FIGURE 4:**
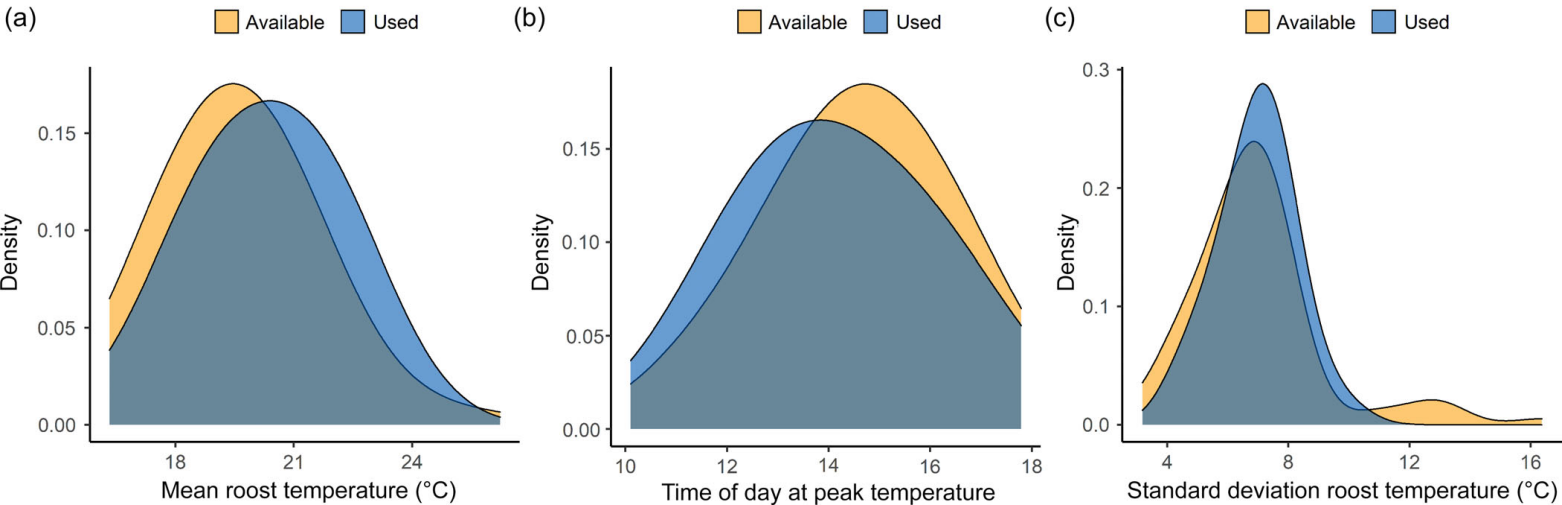
Kernel density plots comparing thermal characteristics within used and available microhabitats: mean temperature (a), time of day at peak temperature (b), and the standard deviation of temperature (c). Blue distributions represent used microhabitats, whereas orange distributions represent available microhabitats. These plots illustrate the results of our binomial model of microhabitat selection. Used microhabitats were slightly warmer on average than available microhabitats, but their distributions largely overlap (a). Temperatures peaked slightly earlier in used microhabitats than available microhabitats, but this was a function of temperatures in warmer microhabitats tending to peak earlier in the day (*r* = −0.19 for the relationship between mean temperature within microhabitats and time of day at peak temperature) and their distributions largely overlap (b). The standard deviation in temperatures within used microhabitats is very similar to the standard deviation in temperatures within available microhabitats, although bats did not use the few microhabitats with very high standard deviations (c). Panel (a) in this figure corresponds to the middle row in Figure 2, and is most closely matched by Figure 2h

**FIGURE 5:**
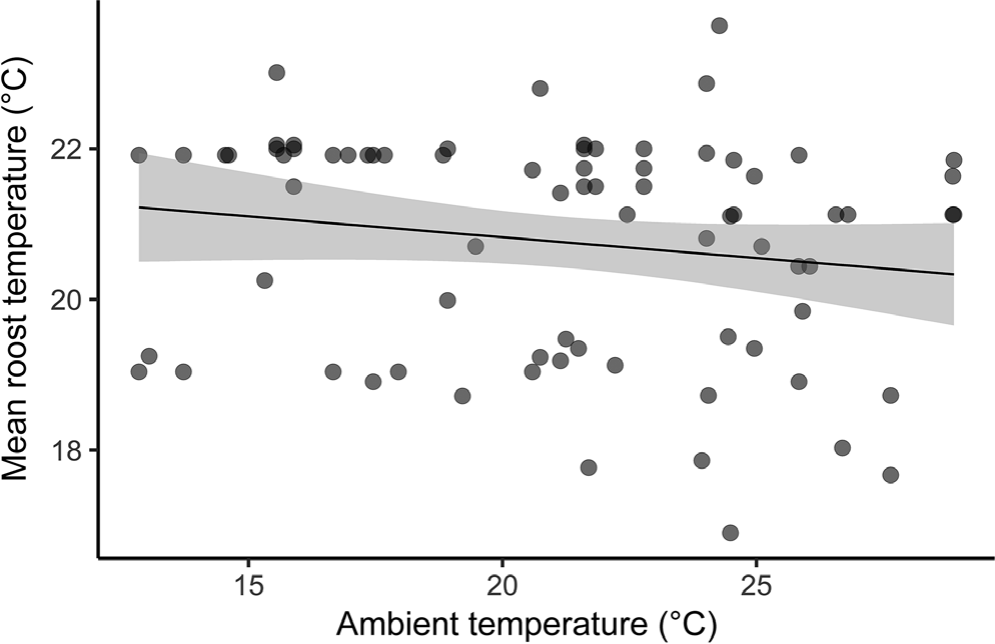
Scatter plot of the relationship between ambient temperature on a given day and mean temperature within used microhabitats. Each point is based on observed data, and represents a microhabitat used for 1 day; some microhabitats (*n* = 14) were used on multiple days and are therefore represented by multiple points on this plot. The line represents the regression line for this relationship and the gray band represents 95% confidence intervals. Ambient temperature on a given day did not influence whether bats used warm or cool microhabitats (*p* = 0.155; *R* ^2^ = 0.03). This figure corresponds to the bottom row in Figure 2, and is most closely matched by Figure 2i

Despite substantial variation in temperatures among microhabitats, we found little evidence that the thermal characteristics of used microhabitats differed from those of available microhabitats (Figure 4). In our model of microhabitat selection, 95% credible intervals for the effect of mean ambient temperature over the course of the day on microhabitat selection did not cross zero (parameter estimate: 0.29; 95% credible intervals: 0.03–0.56), indicating that bats were more likely to select warm microhabitats than cool ones. However, predictive performance was poor (Area under the Curve [AUC]: 0.641) and, overall, used microhabitats (20.3°C) had similar mean temperatures as available microhabitats (19.7°C; Figure 4a). Bats also did not differentiate between microhabitats with temperatures peaking late in the day versus microhabitats with temperatures peaking early in the day (Figure 4b). In our model of microhabitat selection, 95% credible intervals for the effect of the timing of daily peaks in temperature on microhabitat selection crossed zero (parameter estimate: −0.13; 95% credible intervals: −0.38 to 0.11). Overall, used microhabitats (2:10 PM) peaked in temperature at similar times as available microhabitats (2:35 PM). Bats also did not differentiate between microhabitats with stable temperatures and those with more variable temperatures (Figure 4c). In our model of microhabitat selection, 95% credible intervals for the effect of standard deviation in microhabitat temperature over the course of the day on microhabitat selection crossed zero (parameter estimate: −0.18; 95% credible intervals: −0.44 to 0.06). Overall, there was little difference between the standard deviation of temperatures of used microhabitats (6.9°C) and available microhabitats (7.0°C). Finally, there was also no relationship between ambient temperature on a given day and mean temperatures within microhabitats used on that day (*R* ^2^ = 0.03; *p* = 0.155; Figure 5).

## DISCUSSION

The thermal environments in which animals operate strongly influence physiological processes, and can thereby pose substantial challenges in variable environments. How animals overcome these challenges is a central question in animal ecology, but far more research effort has been devoted to understanding the heat‐mitigating behaviors of homeotherms than those of heterotherms. Because heterotherms are not as strongly tied to narrow ranges of body temperature as are homeotherms, the relationships between temperature and microhabitat selection for heterotherms should differ fundamentally from those of homeotherms. Specifically, whereas homeotherms select microhabitats near the thermoneutral zone during periods of inactivity, heterotherms should have less incentive to do so.

We sought to better understand how variation in ambient temperature influences use of daily torpor and microhabitat selection for heterotherms, using a species of bat as a model system. Simulations of energy expenditure in microhabitats that varied in temperature (from 16.9 to 23.6°C on average) revealed that bats can modulate the use of daily torpor to maintain constant energy expenditure over the course of a day over a wide range of temperatures within microhabitats. As a result, microhabitat selection was not driven by temperatures within microhabitats. Our results provide evidence for Prediction Set 3 (no selection) described in the “*Introduction*” section (Figure 2).

The energetic savings associated with daily torpor—particularly at cooler temperatures—are likely to result in microhabitat selection that differs substantially from microhabitat selection by homeotherms. For example, we showed that the use of daily torpor reduces the energetic costs of inhabiting microhabitats that are colder than optimal for homeotherms. If bats were strict homeotherms, the energetic costs of inhabiting cool microhabitats would have been substantially higher (Figure 3), which would probably result in bats selecting warm microhabitats. In contrast, heterothermic bats face little pressure to select warm microhabitats during the active season, even on relatively cool days. For heterotherms, daily torpor does not merely loosen the thermal constraints on habitat selection that homeotherms face at temperatures below the thermoneutral zone, it can entirely mitigate them. Additional studies of the relationships between temperature, torpor use, and microhabitat selection would be valuable for establishing the generality of this finding for other heterothermic species.

Individual traits (e.g., sex, age, and reproductive condition) can alter the energetic costs and benefits of using daily torpor for heterotherms, thereby driving divergence from the pattern demonstrated in this study. For example, microhabitat selection by bats varies by sex, age, and reproductive condition (Elmore et al., 2004; Hein et al., 2008). Whereas male bats in our study did not select microhabitats with specific thermal characteristics, female bats seem to use less daily torpor and prefer warmer microhabitats than males while pregnant or raising young, and females typically aggregate in social maternity colonies rather than roosting solitarily (Hamilton & Barclay, 1994; Kerth et al., 2001; Ruczyński, 2006). Compared with males, then, microhabitat selection by females is likely to be governed more strongly by microhabitat temperature (although social thermoregulation via huddling can influence temperatures within microhabitats more than a microhabitat's physical and environmental characteristics; Pretzlaff et al., 2010; Willis & Brigham, 2007). Further research on the roles of sex, age, and reproductive condition on use of daily torpor by heterotherms (and therefore microhabitat selection by heterotherms) is likely to reveal important context for our findings.

Climate warming increases energy expenditure for many animals, including homeotherms (Humphries et al., 2002; Şekercioğlu et al., 2012). However, the degree to which climate warming will impact heterotherms is poorly understood, largely due to a lack of data on relationships between ambient temperature, torpor use, and thermolability that is needed to accurately model the influence of ambient temperature on heterotherm metabolism (Levesque et al., 2016). Our results indicate that temperature‐dependent use of daily torpor may stabilize energy expenditure, and therefore buffer against the energetic costs associated with variable ambient temperatures. However, most of the energetic savings from heterothermy arise during periods of cold. Increased temperatures due to climate change may therefore reduce the relative energetic benefits of heterothermy compared with homeothermy, as homeotherms experience fewer and milder periods of cold.

Animals that go into torpor for extended bouts—particularly during periods of hibernation—face physiological costs that fall outside the simplified energy balance framework we describe in this paper, including accumulation of metabolic wastes, dehydration, sleep deprivation, and suppressed immune responses (Humphries et al., 2003). In our study, we assumed that these physiological costs did not influence torpor use by bats, and our results suggest that this is true: selecting warmer roosts would allow bats to use torpor less and avoid its physiological costs, but bats did not do this. However, these physiological costs can play major roles in microhabitat selection during hibernation. For example, hibernating bats tend to select the warmest microclimates they can withstand without compromising survival or reproduction, even though doing so increases energy expenditure (Boyles et al., 2007; Czenze et al., 2017; Wojciechowski et al., 2007). Research on torpor behavior and habitat selection during the fall and spring when bats are not fully hibernating but are exposed to colder temperatures and lower resource availability may reveal the conditions in which the non‐energetic physiological costs of torpor use begin to influence microclimate selection.

In warm, dry environments such as our study site, heterotherms often use torpor to reduce water loss in addition to energy expenditure (Bondarenco et al., 2013; Geiser & Brigham, 2012; Nowack et al., 2017). Although we did not account for water loss in our theoretical framing or analyses, water balance could play an important role in driving torpor use (and therefore habitat selection) in bats. However, bats selecting roosts or using daily torpor to minimize water loss should select cooler roosts to reduce evaporation during the day. We did not observe this, indicating that water loss plays a minimal role in roost selection and torpor use in this system.

In conclusion, we showed that a heterothermic bat selected neither warm nor cool microhabitats, because bats can modulate their use of daily torpor to stabilize energy expenditure over the course of a day. Unlike homeotherms, bats face little pressure to select warm microhabitats to avoid heat loss during periods of inactivity; when maintaining a high, stable body temperature becomes energetically costly, bats can calibrate the duration of daily torpor such that energy expenditure stays constant through a wide range of ambient temperatures. Although such finetuning of daily torpor use to stabilize daily energy expenditure is intuitive, it has not been demonstrated in previous studies to the best of our knowledge.

## CONFLICT OF INTEREST

The authors declare no conflict of interest.

## Supporting information


Appendix S1

Data S1

## Data Availability

Data and code (Alston et al., 2022) are available on Zenodo at https://doi.org/10.5281/zenodo.5816672.
