# Supplementary material for: Daily torpor reduces the energetic consequences of microhabitat selection for a widespread bat
Source: Ecology. 2022 Apr 11;103(6):e3677. doi: 10.1002/ecy.3677 (PMC9286574; doi:10.1002/ecy.3677)

Supporting Information. Alston, J.M., M.E. Dillon, D.A. Keinath, I.M. Abernethy, and J.R. Goheen. 2022. Daily torpor reduces the energetic consequences of microhabitat selection for a widespread bat. *Ecology.*

Appendix S1: Supplementary Data

Descriptive Statistics for Rock vs. Tree Microhabitats

During the day, rock microhabitats averaged 20.3˚C (range: 16.6˚ – 24.7˚C) while tree microhabitats averaged 19.1˚C (range: 16.3˚ – 26.2˚C). Daily maximum temperatures within rock microhabitats averaged 25.9˚C (range: 17.9˚ – 38.3˚C), while daily maximum temperatures within tree microhabitats averaged 28.3˚C (range: 21.0˚ – 52.1˚C). Temperatures within rock microhabitats peaked at 1444 hrs on average (range = 1006 – 1748 hrs), while temperatures within tree microhabitats peaked at 1357 hrs on average (range = 1056 – 1659 hrs). Ambient temperature strongly influenced temperatures within microhabitats. Temperatures within rock microhabitats at each hour (in ˚C) followed the equation 7.84 + 0.72*ambient temperature (R^2 = 0.54), while temperatures within tree microhabitats at each hour followed the equation 1.81 + 0.99*ambient temperature (R^2 = 0.62).

Temperatures within used rock microhabitats averaged 20.8˚C (range: 16.9˚ – 23.6˚C) while temperatures within available rock microhabitats averaged 20.0˚C (range: 16.6˚ – 24.7˚C). Temperatures within used tree microhabitats averaged 19.0˚C (range: 17.8˚ – 20.8˚C) while temperatures within available tree cavities averaged 19.6˚C (range: 16.3˚ – 26.2˚C) and temperatures within available spaces under sloughing bark averaged 18.7˚C (range: 16.4˚ – 21.4˚C).

Temperatures within used rock microhabitats peaked on average at 1417 hrs (range: 1106 – 1736 hrs), while temperatures within available rock microhabitats peaked on average at 1500 hrs (range: 1006 – 1748 hrs). Temperatures within used tree microhabitats peaked on average at 1447 hrs (range: 1125 – 1659 hrs), while temperatures within available tree cavities peaked on average at 1411 hrs (range: 1121 – 1615 hrs) and temperatures within available spaces under sloughing bark peaked on average at 1349 hrs (range: 1056 – 1608 hrs).

The standard deviation of temperatures within used rock microhabitats was 6.7˚C (range: 4.3˚ – 10.0˚C), while the standard deviation of temperatures within available rock microhabitats was 6.1˚C (range: 3.2˚ - 11.0˚C). The standard deviation of temperatures within used tree microhabitats was 7.6˚C (range: 6.7˚ - 9.1˚C), while the standard deviation of temperatures within available tree cavities was 8.7˚C (range: 5.8˚ - 16.4˚C) and within available spaces under sloughing bark was 7.7˚C (range: 6.4˚ - 11.0˚C).

There was no difference in ambient temperature between days where rock microhabitats were used and days where tree microhabitats were used (Mann-Whitney U = 287; p = 0.816).

Table S1. Information on daily torpor use by bats tracked during our study, including an ID number for each individual, the dates for which we have data, the mass of bats at time of capture, the timing of torpor entry and exit for morning and afternoon bouts of torpor, the duration of periods of torpor in both mornings and afternoons, and the total duration of torpor across the day.

**Bat ID**	**Date**	**Mass (grams)**	**AM Torpor Start Time (hrs)**	**AM Torpor End Time (hrs)**	**Duration of AM Torpor (mins)**	**PM Torpor Start Time (hrs)**	**PM Torpor End Time (hrs)**	**Duration of PM Torpor (mins)**	**Total Torpor Duration (mins)**
172_063	8/5/2017	6.02	517	1456	579	2013	2055	42	621
172_063	8/6/2017	6.02	451	1210	439	1910	2037	87	526
172_063	8/7/2017	6.02	2245	1557	1032	1840	2044	124	1156
172_904	6/28/2018	6.75	425	733	188	1825	2057	125	313
172_904	6/29/2018	6.75	419	1037	378	1603	2114	277	655
172_904	7/3/2018	6.75	525	944	259	1834	2029	115	374
172_904	7/4/2018	6.75	412	1446	634	1709	2122	253	887
172_904	7/5/2018	6.75	424	1458	597	1930	2043	73	670
172_904	7/6/2018	6.75	511	1016	305	-	-	0	305
172_904	7/7/2018	6.75	438	818	220	-	-	0	220
172_692	7/13/2018	6.92	445	830	225	1936	2043	67	292
172_692	7/14/2018	6.92	435	815	220	-	-	0	220
172_632	7/20/2018	8.04	426	1102	396	1916	2041	85	481
172_753	7/27/2018	8.16	133	2045	1152	-	-	0	1152
172_753	7/28/2018	8.16	2300	2031	1291	-	-	0	1291
172_453	8/4/2018	7.1	449	959	310	1915	2039	84	394
172_784	8/4/2018	7.53	442	1028	346	1951	2023	32	378
172_453	8/5/2018	7.1	459	1156	417	1613	2028	255	672

172_784	8/5/2018	7.53	445	1100	375	1852	2019	87	462
172_453	8/6/2018	7.1	441	916	275	1823	2034	131	406
172_784	8/6/2018	7.53	449	1003	314	-	-	0	314
172_453	8/7/2018	7.1	444	1041	357	-	-	0	357
172_784	8/7/2018	7.53	502	850	228	-	-	0	228
172_063	8/8/2018	6.02	2335	1427	892	1737	2009	152	1044
172_453	8/8/2018	7.1	451	839	228	-	-	0	228
172_784	8/8/2018	7.53	439	852	253	-	-	0	253
172_453	8/10/2018	7.1	456	843	227	-	-	0	227

Fig. S1. An example of raw skin temperature data that we used to delineate bouts of daily torpor. Periods of time in red blocks represent periods of activity (flying, foraging, etc.), periods of time in blue blocks represent periods of daily torpor, and periods in white represent periods of homeothermy or transition between torpor and homeothermy/activity. To delineate bouts of daily torpor, we used the definition suggested in Barclay et al. (2001).

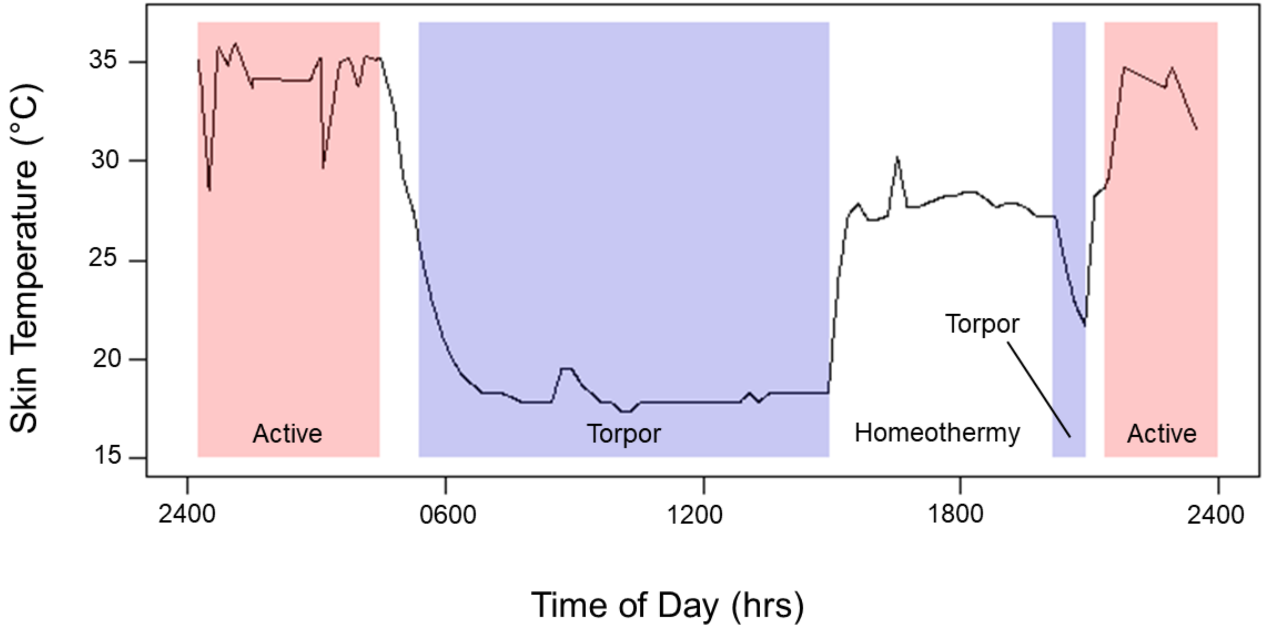

Literature cited

Barclay, R.M., C.L. Lausen, and L. Hollis. 2001. What's hot and what's not: defining torpor in free-ranging birds and mammals. Canadian Journal of Zoology 79:1885–1890.

Fig. S2. Scatter plot illustrating the conditional effect of daily mean ambient temperature on the total duration of bouts of daily torpor during the day. Each point is based on observed data and represents one day. The line represents the regression line for this relationship and the grey band represents 95% credible intervals around this line. Credible intervals for this conditional effect did not cross zero (parameter estimate: -37.4 min; 95% credible intervals: -64.0 – -12.6 min), indicating that bats spent ca. 37 minutes less in torpor per day for each additional 1˚C in daily mean ambient temperature between 0445 hrs and 2100 hrs.

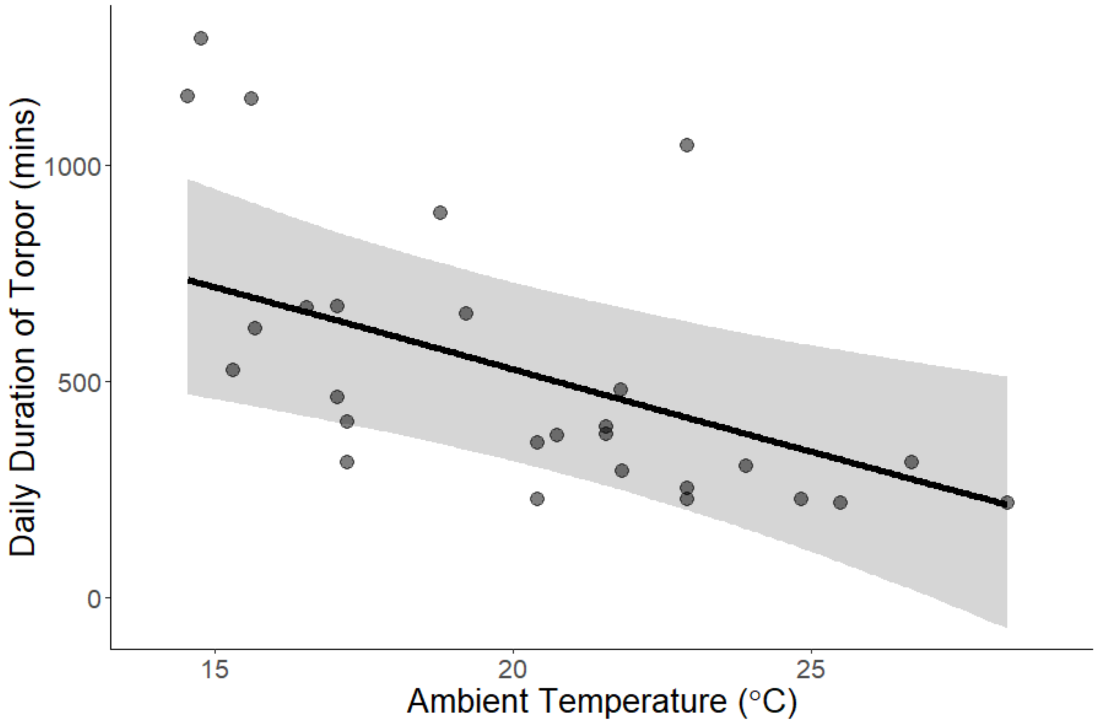

Supplement: Supplementary file 1 — Appendix S1 [file ECY-103-0-s001.pdf]
