# Supplementary material for: Daily torpor reduces the energetic consequences of microhabitat selection for a widespread bat
Source: Ecology. 2022 Apr 11;103(6):e3677. doi: 10.1002/ecy.3677 (PMC9286574; doi:10.1002/ecy.3677)
Supplement: Supplementary file 2 — Data S1 [file ECY-103-0-s002.zip › MetadataS1.pdf]

**JOURNAL PUBLICATION CITATION: Alston, J.M., M.E. Dillon, D.A. Keinath, I.M. Abernethy, and J.R. Goheen. 2022. Daily torpor reduces the energetic consequences of microhabitat selection for a widespread bat. *Ecology.***

---

## Data S1

### R code for “Daily torpor reduces the energetic consequences of microhabitat selection for a widespread bat”

---

## Authors

Jesse Alston
Center for Advanced Systems Understanding (CASUS)
Untermarkt 20, 02826 Goerlitz, DEU
jalston@uwyo.edu

Michael E. Dillon
University of Wyoming
1000 E. University Ave., Laramie, WY 82071, USA
michael.dillon@uwyo.edu

Douglas A. Keinath
US Fish and Wildlife Service
334 N. Parsley Blvd, Cheyenne, WY 82007, USA
douglas_keinath@fws.gov

Ian M. Abernethy
Wyoming Natural Diversity Database
1000 E. University Ave., Laramie, WY 82071, USA
fisher@uwyo.edu

Jacob R. Goheen
University of Wyoming
1000 E. University Ave., Laramie, WY 82071, USA
jgoheen@uwyo.edu

---

## File list (file found within DataS1.zip)

`alston_et_al_myth_roost_code_final.R`

## Description

`alston_et_al_myth_roost_code_final.R` – R code required to reproduce results and figures from "Daily torpor reduces the energetic consequences of microhabitat selection for a widespread bat"
